# Stimuli Responsive Polymeric Systems for Cancer Therapy

**DOI:** 10.3390/pharmaceutics10030136

**Published:** 2018-08-22

**Authors:** Ali Alsuraifi, Anthony Curtis, Dimitrios A. Lamprou, Clare Hoskins

**Affiliations:** 1 Institute of Science and Technology in Medicine, Keele University, Keele ST5 5BG, UK; a.t.y.alsuraifi@keele.ac.uk (A.A.); a.d.m.curtis@keele.ac.uk (A.C.); 2 College of Dentistry, University of Basrah, Basrah 61004, Iraq; 3 School of Pharmacy, Queen’s University Belfast, Belfast BT9 7BL, UK

**Keywords:** stimuli responsive, smart polymer, intelligent polymer, cancer therapy

## Abstract

Nanoscale polymers systems have dominated the revolution of drug delivery advancement. Their potential in the fight against cancer is unrivalled with other technologies. Their functionality increase, targeting ability and stimuli responsive nature have led to a major boom in research focus. This review article concentrates on the use of these smart polymers in cancer therapy. Nanotechnologies have shown potential as drug carriers leading to increased drug efficacy and penetration. Multifunctional smart carriers which can release their payload upon an external or internal trigger such as pH or temperature are proving to be major frontrunners in the development of effective strategies to overcome this disease with minimal patient side effects.

## 1. Introduction

Cancer remains to be one of the most dreadful diseases affecting millions of lives all over the globe. According to the word health report, cancer is the second leading cause of death globally, with over 8.8 million deaths worldwide in 2015; nearly one in six deaths is due to cancer [1]. Cancer is a complex disease and can be summarised as the uncontrolled growth of cells which leads to the formation of a malignant tumour. Not only is it difficult to understand and to treat cancer, it is even difficult to define cancer. Cancer is a wide-ranging term, which used to refer to many different disease states, all characterised by unusual cell growth. Tumour medications typically depend on surgery, radiotherapy and chemotherapy or combinations thereof. To a lesser degree, hormone treatment, hyperthermia, immunotherapy and undifferentiated cell help are also utilised [2]. However, there are many side effects from extant treatments, and new therapies are urgently required that specifically target carcinoma cells without damaging healthy tissue.

Chemotherapeutics by the injection of free drug into circulating blood used in traditional cancer treatment is generally limited because of the short blood circulation time as well as the very low specificity of drug action. Majority of chemotherapeutic agents have aqueous solubility and thus pose a challenge in delivering those drugs effectively. Most of the current formulations have organic solvents, a high concentration of surfactants or other co-solvents which are toxic to the human being upon chronic exposure. As a result, the main disadvantage is the exposure of healthy tissues to the drugs, resulting in a risk of harmful damage or even cellular mortality [3]. Additionally, undesirable patient side effects are experienced such as suppressed immune system, hair loss, nausea, fatigue, etc. Typically, the site of action is exposed to only a low concentration of the therapeutic agent, whilst the remainder is distributed systemically throughout the body. This unavoidable circulation into solid organs and tissues and the lack of targeting, which limits the dose concentration that can be given, prevents these medications from attaining the therapeutic index at the desired site of action [4]. Targeted drug delivery seeks to direct the drug of choice to the tissues of concern whilst decreasing systemic circulation, thus evading the host’s immune systems and hindering widespread delivery, particularly in the liver and spleen [5].

The cost as well as time required to develop new drug molecules is large hence, large pharmaceutical companies are encouraging researchers to use formulation strategies to improve the safety and biological relevance of “old” drugs; teaching “old” drugs new tricks. Drug delivery in a rate-controlled, targeted manner is very attractive and is the focus of much attention [6]. The development of effective therapies for the treatment of cancer relies on the development of effective carriers that are nontoxic, able to carry a significant payload of the anticancer molecule to the appropriate cells, with high accuracy, to achieve effective cell death, and which allow combination therapeutic platforms. The main aim of any targeted drug delivery system is to prevent the drug release on administration and control the delivery of the active ingredient. Controlled delivery systems avoid the harmful effects that usually occur when the bolus injection is administered, resulting from of the high plasma drug concentration. Using targeted delivery, tumours can be exposed to therapeutic levels of the medication for a continual period with decreased systemic toxicity. Drug inclusion in a polymer formulation allows for tailoring of release kinetics to achieve the most efficacious delivery regimen [7]. The physicochemical and pharmacokinetic properties of the drug play a key role in the design of such a drug delivery system.

Progress in drug design has led to the development of new peptides, proteins, and drug molecules that can be used for the treatment of cancer. However, the limited ability to selectively deliver these molecules specifically into cancer cells, at well-defined dosing regimens and without invoking drug-resistance remains a significant challenge. Poor aqueous solubility or dissolution of medications has become one of the most significant difficulties in drug delivery. Approximately 70% of new drugs under investigation possess poor water solubility and, hence, poor bioavailability and delivery difficulties. Many methodologies have been utilised to enhance water solubility, for example, the use of co-solvents, micronisation and nanonisation, amorphous solid dispersions (ASDs), co-crystal formation, surfactants, complexation utilising cyclodextrins and the use of polymers [8].

The use of drug delivery systems such as nanoparticles loaded with drugs and anticancer peptides to enhance the therapeutic effect of anticancer agents and reduce systemic toxicity has been widely investigated. Amphiphilic polymers have proven to be effective technologies in the solubilisation of lipophilic active pharmaceutical ingredients. These systems are composed of a hydrophilic and hydrophobic moieties which spontaneously aggregate into core–shell self-assemblies upon exposure to aqueous environments [9]. The first reports of amphiphilic polymers for drug delivery were polymer drug conjugates [10]. In these systems, the lipophilic drug molecules were conjugated directly and irreversibly onto hydrophilic polymer chains [11]. Once aggregated in aqueous environments, the drug moieties clustered inside the core of the self-assemblies formed, thus “shielding” them from the polar environment [12]. Leading on from the polymer drug conjugates, a class of amphiphilic polymers were developed [13]. These systems were formed from multiple architectures, such as block copolymers and graft polymers [14]. Similar to polymer–drug conjugates before them, these systems also formed self-assemblies. The advantage of these systems over the polymer drug conjugates was that the drug could be added independently to the polymer and also released “easily” [15]. Here, when mixed with the amphiphiles in aqueous environments, the lipophilic drug molecules become encapsulated within the hydrophobic core of the self-assemblies due to a reduction in Gibbs’ free energy [16]. As these systems exist in dynamic equilibrium, the drug release in native form is achieved. Numerous studies have been reported concerning the manipulation of polymer architecture or composition in order to tailor the level of drug encapsulation and release rate of this class of polymers [17,18,19,20,21]. The concept of drug encapsulation into the hydrophobic core of aggregates is not new; this concept has been exploited in the pharmaceutical industry for many years through the use of surfactants [22]. However, encapsulation within polymers offers numerous advantages over the use of surfactants, including highly reduced excipient to drug ratios which result in more efficient, stable and cost-effective systems in addition to a reduction in excipient toxicity [23]. Additionally, these high molecular weight polymers form nano-scale self-assemblies, which can preferentially accumulate inside tumour tissue due to the exploitation of the enhanced permeability and retention (EPR) effect [24]. This is due to the leaky vasculature of tumorous tissue which malforms under rapid proliferation. Nano-sized particles can permeate these leaky capillaries and accumulate in the tumour tissue [25]. Here, they do not re-enter the capillaries due to poor lymphatic drainage and, hence, a degree of passive targeting is achieved [26]. The use of degradable polymers has been successfully reported in drug solubilisation [27]. Typical polymer backbones used as solubility enhancers are poly(amino acid) [26], poly(ethylene oxide) [28], poly(ethylenimine) [29], poly(allylamine) [30] and chitosan [31].

**Figure 1:**
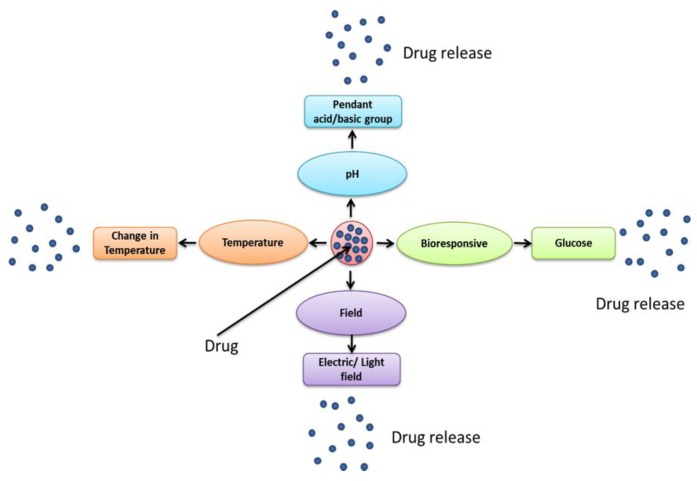
Different types of smart polymeric drug delivery systems for controlling drug release.

Advancements in polymer chemistry have led to the fabrication of smart polymer systems. These systems enable further control over the physicochemical behaviour of the polymers which is highly desirable in the targeted delivery of chemotherapies. Smart polymeric materials or environmentally-responsive polymers, are polymers that respond to different stimuli or changes in the environment, for example temperature, pH, electric and magnetic fields, light intensity, biological molecules, etc. [32,33] (Figure 1). These stimuli can result in many responses such as a change in colour or transparency, becoming conductive, increased permeability to water or changing shape [34,35]. In all instances, these systems may be formed by, either drug encapsulation via hydrophobic–hydrophobic interaction, physical conjugation onto the polymer backbone creating an amphiphile or, indeed, surface functionalisation of a preformed nanoparticle.

Table 1 summarises examples of stimuli responsive polymers used in cancer therapy along with their corresponding stimulus.

## 2. pH Responsive Polymers

Recently, pH-sensitive polymers have been gaining attention for applications in drug delivery [49] and cancer therapy [50].

pH-responsive polymers are polyelectrolytes that incorporate contain weakly acidic or basic ionisable groups with pKa values 3 to 10 within their structure [51]. These groups either accept or donate protons in response to changes in environmental pH. The pendant acidic or basic groups such as carboxylate, sulfonate and amino groups on polyelectrolytes demonstrate a change in ionisation state as a function of pH. The alterations in structural and other properties such as solubility, surface activity, and chain conformation have been explained by changes in the ionisation, whereby specific polymer groups switch between a neutral and charged state [52]. These unique properties of pH-responsive polymers have sparked particular interest in their use in drug delivery applications based on the fact that the human body presents a range of pH along the gastrointestinal tract and also in some specific segments of tumours, which provide environmental stimuli for responsive drug release (Table 2).

**Figure 2:**
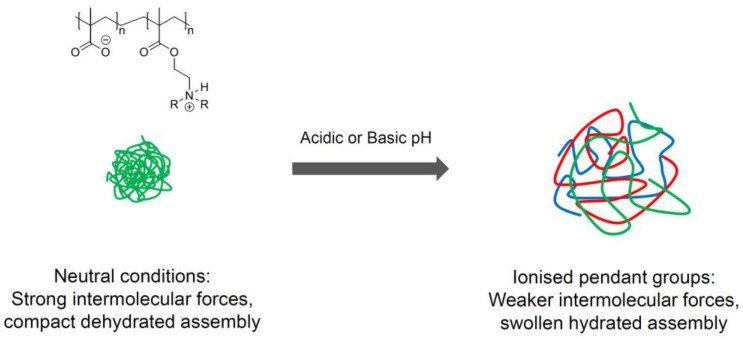
Representation of the switch between a neutral and charged state of pH-responsive polymer particles.

An increase in the degree of ionisation contributes to electrostatic repulsion between charged groups and, thus, swelling of the polymer occurs at which point drug release can be achieved [53,54,55] (Figure 2).

This offers potential in drug targeting, since the polymer assemblies can be directed to respond to certain pH changes within tissues or cellular compartments due to their localised pH. Several different mechanisms can control the release of encapsulated drugs from a pH-responsive polymer including diffusion controlled, swelling controlled, and chemically controlled mechanisms [56].

In addition to the “normal” intracellular pH range, cancerous cells create more lactic acid than normal cells due to their increase in glycolysis and proton-pump action. The lactic acid is discharged into the extracellular regions, prompting a lower extracellular pH (pH 6.5–7.2) compared to the bloodstream and normal tissues (pH 7.4) [57,58]. Hence, vehicles can be developed which undergo swelling or shrinking at this pH, thus disrupting their micellar structure and resulting in drug release. This combination of ability to passively target cancerous tissue as well as the stimuli triggered drug release of these systems offers an exciting future for cancer therapy. Most of the stimuli responsive systems in the literature are based on acrylates or similar structures and derivatives, with poly(acrylic acid), poly(methacrylic acid) and poly(*N*,*N*-dimethyl aminoethyl methacrylamide) being the most commonly used [55].

### 2.1. Poly(acrylic acid)

**Figure 3:**
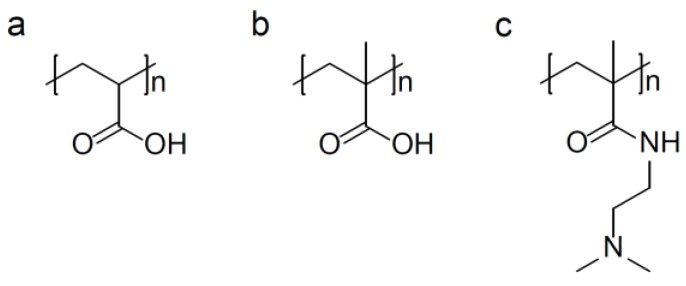
Chemical structures of common pH responsive polymers: (**a**) poly(acrylic acid); (**b**) poly(methyacrylic acid); and (**c**) poly(*N*,*N*-dimethyl aminoethyl methacrylamide).

Polyacrylic acid (PAA) (Figure 3a) is a weak anionic polyelectrolyte, whose degree of ionisation is dependent on solution pH. This confers the ability to absorb and retain water and swell to many times their original volume. This phenomenon is exploited in pH controlled release of drug compounds.

Lee and colleagues developed a series of doxorubicin-loaded polymer-caged nanobins (PCNDXR) for breast cancer treatment [63]. The novel formulation was based on a liposomal encapsulation of doxorubicin with cross-linked cholesteryl terminated PAA deposited onto the surface. The mechanism of release was via polymer shrinkage leading to vesicle collapse at reduced pH (pH 5). The anticancer abilities of the novel formulations were evaluated in vivo in murine xenograft models of triple-negative breast cancer. The study showed that, by varying the degree of cross-linking in the polymer cage, the in vivo circulation lifetime of the nanocarriers could be tuned. The study demonstrated that the PCNDXR could effectively inhibit tumour growth and, importantly, the PCNDXR was well tolerated by mice. The formulation resulted in a reduction in systemic toxicity compared with the free drug [63].

### 2.2. Poly(methacrylic acid)

Similar to PAA, Poly(methacrylic acid) (PMAA) (Figure 3b) is a commonly used polymer with a pKa around 4.8. At neutral pH, the methacrylic acid groups are almost entirely deprotonated. PMAA acts as a polyelectrolyte and acts as a sponge where it readily absorbs and retains water. However, these properties are strongly pH dependant and reversible. Hence, the pH dependant nature makes PMAA ideal as a stimuli responsive drug carrier.

Recently, a chitosan (CS) grafted with PMAA and with graphene oxide (GO) incorporated into the structure to form CS-g-PMMA/GO was reported [64]. The CS-g-PMAA/GO system demonstrated 93.8% drug loading and 78.6% drug encapsulation efficiencies for doxorubicin (DOX). The pH responsive nature of the formulation was determined as measured by drug release. The authors reported that the CS-g-PMAA/GO showed the highest drug release values at pH 4, with negligible release quantified at pH 7.4. Cytotoxicity of the formulation was measured via MTT assay on MCF7 breast cancer cells after 48 h. The results showed that the free drug possessed greater cytotoxicity compared with the formulation. However, due to physicochemical as well as biological issues, the use of free DOX is not recommended; hence, this system could be used to combat or reduce the side effects of DOX if release of the therapeutic payload could be altered to improve the efficacy [64].

### 2.3. Poly(N,N-dimethyl aminoethyl methacrylamide)

As with the other acylamides discussed, the sponge-like pH sensitive electrolyte properties of Poly(*N*,*N*-dimethyl aminoethyl methacrylamide) (PDEAEMA) (Figure 3c) also make it ideal as a stimuli responsive polymer for release of pharmaceuticals. Chen and colleagues reported the use of novel pentablock-poly(ethyleneglycol)-b-(poly(2-(diethylamino)ethylmethacrylate)-b-poly (hydroxy ethyl methacrylate)-g-folic acid)2 [PEG-b-(PDEAEMA-b-PHEMA-g-FA)2] for anticancer therapy [65]. Here, the polymeric micelles were loaded with DOX. The drug loaded micelles were below 120 nm in size, with 48% entrapment efficiency. The micellar drug release was determined; these data showed that greater DOX cumulative release was observed at pH 5.0 (~90%) compared to pH 7.4 (~20%), due to protonation of the tertiary amino groups. In vitro cytotoxicity was determined in human hepatocellular carcinoma cells (HepG2) after 24 h exposure. These studies showed that the cytotoxicity of the formulation was similar to that of the free drug. However, cellular uptake studies showed that the loaded formulations resulted in more rapid cellular internalisation compared with the free drug which would result in faster tumour treatment in vivo [65].

### 2.4. Other pH Responsive Systems

Hu and colleagues prepared a pH-sensitive polymeric vehicle, in which a poly(l-histidine)-b-short branched poly(ethyleneimine) (PEI) was conjugated to paclitaxel (PTX) [66]. Here, they reported drug release occurring at a pH similar to the tumour environment. They reported a large drug uptake and increase in efficacy after pH-mediated release. The cationic micelle surface was shielded by electrostatically complexing it with a negatively charged methoxy(polyethylene glycol) (mPEG) (2 kDa)-b-polysulfadimethoxine (4 kDa) (mPEG-b-PSDM) at pH 7.4. Unshielded micelle at pH 7.4 and deshielded micelle at tumour extracellular pH were readily taken up by two wild types of human cancer cell lines, MCF-7 breast adenocarcinoma and SKOV-3 ovarian carcinoma, while the uptake of the shielded micelle at pH 7.4 was minimal [66].

Zhang and colleagues constructed a series of pH-responsive micelles via conjugating PEG onto a farnesylthiosalicylate derivative FTS-hydrazide (FTS-H), using a hydrazone linker (forming PHF-2) [67]. The linker cleavage characteristics could be tailored by varying the length of the carbon chain or the electron withdrawing groups in the polymer structure. FTS-H has been shown to possess an inherent chemotherapeutic effect. The pH-responsive breakdown of the micelles via hydrolysis in both neutral and acidic conditions was measured. The PHF-2 micelles were found to undergo hydrolysis in acidic conditions whilst remaining intact at neutral pH. Cytotoxicity studies on prostate cancer (PC) cells showed that the micelles retained the inherent toxicity of FTS-H. The micelles were loaded with paclitaxel and in vitro studies demonstrated that the novel formulation possessed enhanced antitumor activity compared with the free drug. This study demonstrates a combined effect for chemotherapy, and further in vivo studies must be performed to verify the in vitro data [67]. 

Kang and colleagues reported the development of a pH-sensitive methoxy poly(ethylene glycol)-4β-aminopodophyllotoxin (mPEG-NPOD-I) via an imine linkage [68]. The mPEG-NPOD-I micelles served as a polymer drug conjugate resulting in significantly faster NPOD release at reduced pH of 5.0 and 4.0 compared with physiological pH. The ability to deliver the NPOD into adenocarcinomic human alveolar basal epithelial cells A549, cervical adenocarcinoma Hela cells, and hepatocellular carcinoma HepG2 cells was investigated. These studies showed that the polymer–drug conjugate delivered NPOD to the nuclei of the tumour cells and led to enhanced cytotoxic effect in all three cell lines compared with the free drug. The IC_50_ for the formulation was one order magnitude lower than that of the free drug. The polymer–drug conjugate was also loaded with paclitaxel (PTX) with drug-loading efficiency of 57%. The PTX loaded formulation exhibited pH-triggered rapid release profiles as expected, and an elevated cytotoxicity was observed compared to the unloaded PEG-NPOD-I [68].

## 3. Thermally Responsive Polymers

Temperature responsive polymeric micelles for chemotherapy are the most extensively studied to date. Due to the high rate of proliferation, cancerous cells possess higher metabolic rates compared to normal tissues. As a result, the intratumoral environment is at a higher temperature of 40–44 °C as compared to normal, healthy tissue [69]. It is this subtle change in temperature within the tumour microenvironment which is ideal for the exploitation of thermo-responsive drug release.

Generally, the solubility of most substances will increase with increased temperature. Smart polymers have been developed which exhibit either an increase or a decrease in solubility in response to a change in temperature. This unique property has resulted in a great focus on thermo-responsive polymers as modern, highly controllable drug delivery systems. Temperature responsive polymers display a fine hydrophobic–hydrophilic balance in their structure which is switchable over small temperature ranges. These adjustments result in bond contraction or stretching, forming new conformations around the hydrophobic moiety and the hydrophilic polymeric chains within the fluid media. The conformational change of the polymer is dependent upon the physical condition of the chains. In the event that the macromolecular chains are straight and solubilised, the arrangement will change from mono-phasic to bi-phasic due to the polymer precipitation upon initiation [70]. This change in polymer structure can be exploited for triggered drug release.

In general, thermo-responsive polymers can be categorised into two groups according to either their lower critical solution temperature (LCST) or their upper critical solution temperature (UCST) properties.

**Figure 4:**
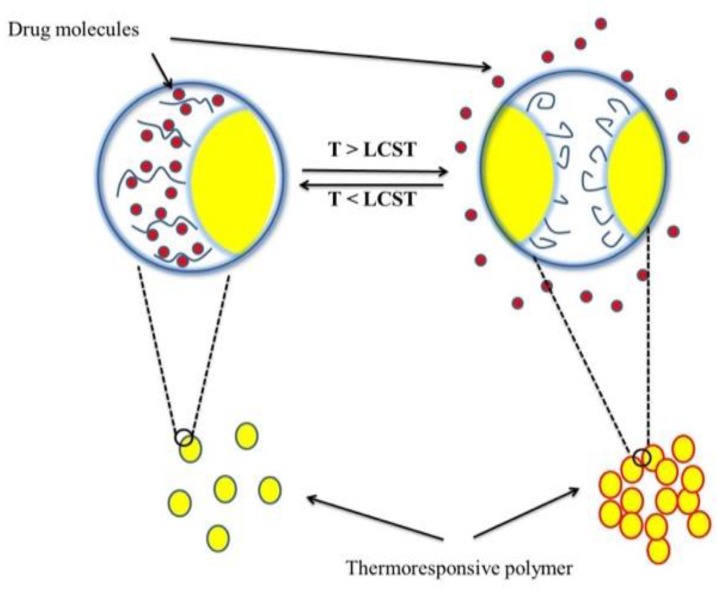
Schematic illustration of the LCST-type phase transition.

Almost all applications of these polymers rely on sudden solubility changes in aqueous environments at the LCST. The rapid change in solubility of thermo-responsive polymers around the LCST can be attributed to the considerable hydrogen bonding interactions with neighbouring water molecules and limited intra/inter-molecular hydrogen bonding between polymer molecules (Figure 4). At increased temperatures, hydrogen bonding between the polymer and water break down and intra- and intermolecular hydrogen bonding/hydrophobic interactions dominate above the LCST, which results in a transition in solubility [71].

There are two important characteristics of thermo-responsive polymers: (1) the possibility to tune their LCST; and (2) sharp phase transitions in both heating and cooling processes. Dehydration and rehydration of the polymer chains are affected by the sharpness in phase transitions, which rely on the balance of interactions between polymer chains and the aqueous solvent, and between the polymer chains themselves [72].

Some of temperature sensitive polymers possessing pharmaceutical relevance are shown in Table 3.

### 3.1. Poly(hydroxypropylmethacrylamide)

**Figure 5:**
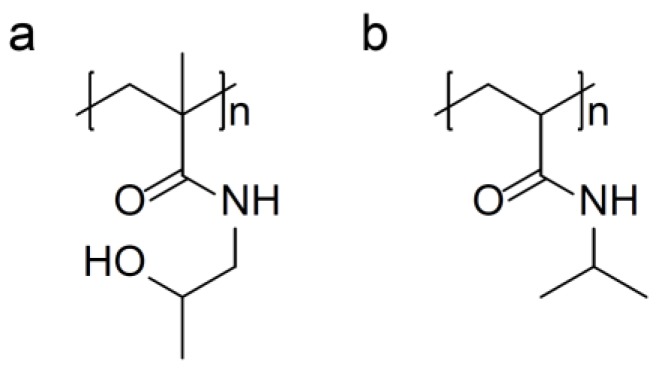
Chemical structure of commonly used thermos-responsive polymers: (**a**) poly(hydroxypropylmethacrylamide); and (**b**) poly(*N*-isopropylacrylaminde).

Perhaps the most successful polymeric systems which have been used for passive drug targeting are *N*-(2-hydroxypropyl) methacrylamide (HPMA) (Figure 5a) based copolymers [81]. Poly(*N*-(2-hydroxypropyl) methacrylamide) (pHPMA) possesses desirable properties as a drug carrier, such as non-immunogenicity, biocompatibility and the possibility for functionalisation. These properties have resulted in a wide range of pharmaceutical and biomedical applications as well as in research into micelle technology [82]. pHPMA self-assemblies have also been used in the design of hybrid block and grafted HPMA copolymers that self-assemble into smart hydrogels [83].

HPMA copolymers conjugates incorporating anticancer drugs have demonstrated significant antiproliferative effects in cancer cells and tumour retardation in vivo. Intending to enhance the result of prostate cancer drugs by focusing on cancer stem cells (CSCs), blockage of the hedgehog (Hh) signalling pathway (an essential pathway included in stem cell self-renewal) by cyclopamine leads to long-term protection against prostate cancer relapse, strongly suggesting the link between the Hh pathway and prostate CSCs [84]. Zhou and colleagues prepared a water-soluble macromolecular delivery system focused around a HPMA copolymer, and assessed the CSC inhibitory impacts of the HPMA copolymer–cyclopamine conjugate in an in vitro prostate cancer epithelial cell (RC-92a/hTERT) model. The bioactivity of cyclopamine was present after conjugation to the polymer. Moreover, HPMA polymer-conjugated cyclopamine indicated increased potency of CSC on RC-92a/hTERT cells as assessed by diminished stem organism marker interpretation and CSC reasonability [84].

Buckway and colleagues showed the breaking down of hyaluronic acid by treating CAPAN-1 xenograft tumours in athymic nu/nu mice with *N*-(2-hydroxypropyl) methacrylamide (HPMA) copolymers radiolabelled for single photon emission computerised tomography (SPECT) imaging [85]. Results showed that tumour targeting can be accomplished in vivo after treatment with hyaluronidase. This methodology resulted in improved drug delivery of polymer–peptide conjugates to solid tumour [85].

Laga and colleagues reported the development of diblock copolymers based on HPMA conjugated with pirarubicin (PIR) [86]. The thermo-responsive nature of the amphiphile created resulted in a coil-globule transition at elevated temperatures. The in vitro cytotoxicity and intracellular trafficking of the novel formulation, as well as drug release of PIR inside the cells was investigated in human colon adenocarcinoma (DLD-1) cell lines. The data showed that the novel HPMA conjugates showed great promise for clinical translation; however, further work is needed to elucidate the in vivo potential and fate [86].

### 3.2. Poly(N-isopropylacrylaminde)

Poly(*N*-isopropylacrylamide) (PNIPAM) (Figure 5b) and its derivatives are well known as the most common thermosensitive polymers, which show phase separation at a LCST around 32 °C in aqueous solution. Above the LCST, it experiences a sharp coil-to-globule transition to form inter- and intrachain association, bringing about hydrophobic accumulation and precipitation from solution, while, under this temperature, PNIPAM is hydrophilic and exists as separate polymers chains which reside in a coil conformation [87].

PNIPAM has been widely used to fabricate temperature sensitive hydrogels as a result of this reversible phase transition. A PNIPAM hydrogel can absorb water and exist in a swollen state beneath the LCST. Nonetheless, it experiences an unexpected and dramatic shrinkage in volume once the environmental temperature is raised above the LCST. PNIPAM is widely used in controlled drug release due to these favourable characteristics [88].

PNIPAM has sharp and reversible phase transition at a temperature near to body temperature because of the secondary amide group and a hydrophobic isopropyl group. Studies have shown that, during the coil-globule transition of PNIPAM, there was a reduction in some hydrogen bonding between C=O and H_2_O, and that, additionally, the C=O groups formed another type of hydrogen bond between C=O and HN [89].

Contreras-García and colleagues grafted poly(propylene) (PP) by *N*-isopropylacrylamide (NIPAAm) and *N*-(3-aminopropyl) methacrylamide hydrochloride (APMA) by using γ-irradiation to obtain polymers that are stimuli-responsive under physiological conditions [90]. The team reported that, when they used 1 M NIPAAm:0.5 M APMA, around physiological temperature, the copolymer showed rapid and reversible transitions showing a LCST, while with higher content of APMA the hydrophilicity of PP-g-(1NIPAAm-r-1APMA) becomes higher, which prevented the grafted polymer from shrinking [90]. Another report from Li and colleagues referred to using reversible addition-fragmentation chain-transfer polymerisation to prepare the hydrophilic block copolymer poly(*N*-(3-aminopropyl)methacrylamide hydrochloride)-b-(*N*-isopropylacrylamide) (PAMPA-b-PNIPAM) [91]. The researchers found that, when the solution temperature is increased, these block copolymers self-assemble into vesicles, the size and size distribution of the vesicles being influenced by the heating rate and the solution concentration [91].

Thermo-responsive polymeric materials can potentially elicit a therapeutically effective dose without unacceptable adverse effects. More recently, several studies have confirmed the ability of a thermo-responsive polymeric micelle to release its payload into the surrounding environment by relatively small differences in temperature. In one report [92], anticancer agent methotrexate was used as a model drug. Methotrexate is a poorly soluble drug which triggers severe patient side effects, thus its formulation is desirable to improve both clinical efficacy and therapeutic safety. To address these problems, Sun and colleagues loaded methotrexate into poly(*N*-isopropylacrylamide-co-acrylamide)-b-poly(n-butyl methacrylate) block copolymer micelles [92]. The ultimate goal was to produce a stimuli-responsive polymer formulation capable of on/off switching of drug release and spatial specificity. Sun’s group reported that the thermally triggered release of methotrexate was observed in vitro. Additionally, this exciting work showed that the cytotoxic activity of methotrexate-loaded micelles was considerably increased in combination with hyperthermia on Lewis lung carcinoma cells, thus proving their thermo-responsive nature [92].

The use of NIPAM is not restricted to micellar studies. Reports have shown that DOX and camptothecin (CPT) could be loaded into shape transformable amphiphilic NIPAM scaffolds [93]. The in vitro drug release studies revealed that the DOX loaded scaffolds showed a selective release to deliver 90% of loaded drug at the cancer tissue temperature (40–43 °C) as compared to that of normal body temperature (37 °C, <10%). The kinetics of DOX release at the cancer tissue temperature indicated that the formulation followed non-Fickian diffusion kinetics [93].

## 4. Light Responsive Polymers

The main advantages that make light, ultraviolet (UV) or visible, one of the most desired external stimuli is that the drug delivery systems are inexpensive and easily controllable or modulated. When a light source with appropriate wavelength is applied to polymers or hydrogels, which contain photosensitisers such as azobenzene, stilbene, and triphenylmethane, the chromophore absorbs the light, resulting in an increase in local temperature. As a result, the polymer or hydrogel changes its degree of swelling behaviour in response to this temperature change [94]. One of the major limitations in the use of light stimuli-responsive polymer in drug delivery systems is the leakage of non-covalently-bound chromophores during swelling or shrinking of the system which leads to an inconsistent and slow response of the system towards the stimulus. With a second drawback being the inherent toxicity before light irradiation [95].

Jiang and co-workers described the synthesis of diblock copolymers containing hydrophilic poly (ethylene oxide) (PEO) and a hydrophobic poly(methacrylate) bearing pyrene pendant groups [96]. The self-assembly of amphiphilic block copolymers bearing these groups in water led to the formation of micelles, which can be dissociated by light. UV light irradiation of the micellar solution triggers the cleavage of the photo-responsive pyrene moieties, generating a hydrophilic poly (methacrylic acid). As a result of the cleavage, the polymer micelles completely dissociate [96].

Recently, a photo-responsive lipid–polymer hybrid nanoparticle system consisting of three distinct functional components has been reported [97]. It is comprised of: (i) a poly (d,l-lactide-co-glycolide) (PLGA); (ii) a soybean lecithin monolayer; and (iii) a photo-responsive polymeric shell with anti-biofouling properties. The PLGA was used to encapsulate DOX while the soybean lecithin monolayer acts as an active barrier to prevent drug leakage. Furthermore, the nanoparticle stability under light irradiation was enhanced by the photo-responsive polymeric shell. The in vitro result indicated that 76% of encapsulated drug was released upon light irradiation as compared to 10% release without light irradiation. The results obtained from confocal microscopy and flow cytometry support the light-controlled drug release behaviour inside the cancer cells [97].

## 5. Multi-Responsive Polymers

Some smart polymeric systems have been devised to respond to more than one stimulus. These polymeric systems could present a unique opportunity to fine tune their response to each stimulus, individually rendering them “super-intelligent”. A pH-responsive nanophotomedicine (pH-NanoPM) was synthesised by Park and colleagues for targeted photodynamic therapy (PDT) [98]. It was reported that the pH-responsive NanoPM was fabricated by self-assembly of a pH-responsive polymeric photosensitiser (pH-PPS) containing pH-cleavable mPEG (pH-C-mPEG) with a size of ~110 nm. They also reported that pH-NanoPM showed improved cellular internalisation at acidic tumour pH compared to normal pH when exposing HeLa human cervical cancer cells, which led to a remarkable cancer cell killing effect [98]

Ryskulova and colleagues investigated the potential of a pH and temperature dual-responsive system comprised of poly(phenylene vinylene)s (PPVs) having both carboxylic acid and methoxyoligoethylene glycol pendant groups [99]. The fluorescence intensity is affected by change pH in a range from 3 to 10 and temperatures from 10 °C to 85 °C [99].

In recent years, the combination of stimuli-responsive polymers and metal nanoparticles into a single platform has attracted much consideration due to their unique properties. Zhou and colleagues reported that a selenium-containing polymer combined with a drug could be used successfully for multi-stimuli responsive drug release [100]. They prepared micelles of selenium-containing PEG with redox-triggered properties and metal-organic frameworks (MOFs) with pH-triggered properties in drug delivery systems. They concluded that the cores easily collapsed in the presence of redox agents whilst the shell can breakdown only under low pH conditions [100].

Magnetic nanoparticles (MNPs) and gold nanoparticles can be triggered by internal or external stimulus which leads to internal changes to the polymeric network, allowing for a versatile stimuli-responsive system [101,102]. Wadajkar and colleagues reported the development of a temperature responsive system based on magnetic nanoparticles coated with poly(*N*-isopropylacrylamide-acrylamide-allylamine) (PMNPs) [103]. The particles were surface decorated with prostate cancer-specific R11 peptides to allow active targeting and imaging in prostate cancer cells. The nanoparticles created possessed a LCST of 40 °C. Magnetic characterisation data confirmed that the nanoparticles could be manipulated using a magnetic field and retained their superparamagnetic properties after surface coating and decoration. In vitro studies showed that R11 decorated PMNPs were biocompatible in human dermal fibroblasts and normal prostate epithelial cells after 24 h of exposure. The nanoparticles were internalised into PC cells and LNCaP in a dose-dependent manner. The in vivo studies showed that the nanoparticles accumulated at significantly higher levels in the tumour tissue compared with the other vital organs. The authors concluded that their results indicate the great potential of this novel formulation for the targeting and monitoring of prostate cancers as well as for diagnostic and therapeutic applications [103].

## 6. Conclusions and Future Perspectives

The fast-developing science of nanotechnology is one of many areas that are expected to have a significant impact on medicine and how medicine is delivered. There are several varieties of particles available, however, smart polymers have shown potential in cancer therapy, and in their use as drug solubilising agents and stabilisers, for offering controlled drug release, as multifunctional platforms for stimuli-responsive release or use in image guided therapy. Due to the relative age of these technologies, few have progressed into the clinic, however it is estimated that exponential clinical exploitation will be evident within the coming two decades. As more and more complex systems are developed, this drives forward the potential for targeted therapies in cancer patients. It is estimated that not only more effective therapies and treatments resulting in improved patient outcomes will be developed, but also more cost effective and affordable therapies will come to the market. The reduction of patient side effects from circulation of highly toxic agents within the bloodstream will also cut down patient–practitioner consultations and accelerate patient recovery. Polymeric nano-systems have a great potential to be used as vehicle for the treatment of cancer with least side effects. The next 10–20 years are looking bright for such patients and ultimately these technologies can be translated into other clinical conditions. As scientific advancement pushes forward these technologies are also being translated to other disease states, sectors and applications. The key to the future of nanotechnology is consistent characterisation and stability regulation and improved knowledge of toxicology surrounding their use in vivo and environmentally. Often polymers get a bad reputation in the press from the very timely topic of plastic accumulation in the environment. As such, careful consideration does need to be paid to the breakdown and longevity of these nano-scale polymeric systems to ensure their medical advancements do not result in long term medical or environmental downsides. Once these barriers are overcome, it is likely that these new systems will dominate the medical market within the next 20–30 years.

## Figures and Tables

**Table 1:** Various stimuli and responsive materials.

Environmental Stimulus	Responsive Material	Reference
Temperature	*N*-(2-hydroxypropyl)methacrylamide) (HPMA), Poly(*N*-isopropylacrylamide) (PNIPAAM), Poly(2-isopropyl-2-oxazoline) (PiPOx)	[36,37,38,39]
pH	Poly(acrylic acid), poly(methacrylic acid) (PMAA), poly(ethylene imine), poly(l-lysine), and poly(*N*,*N*-dimethyl aminoethyl methacrylamide)	[40,41,42,43]
Temperature and Light	Modified poly(acrylamide)s	[44]
Electric field	poly(vinyl alcohol) and poly(acrylic acid-co-2-acrylamido-2-methyl propyl sulfonic acid)	[45]
Ultrasound	poly(lactic acid-co-glycolic acid)	[46]
Temperature and electric field	Poly(pyrrole)	[47]
Temperature and pH	poly(*β*-amino ester)	[48]

**Table 2:** Table showing the localised pH in different tissues and cell compartments [59,60,61,62].

Tissue/Cellular Compartment	pH
Blood	7.35–7.45
Pancreas	8.0–8.3
Bile	7.8
Intestines	7.5–8.0
Saliva	6.0–7.0
Colon	7.0–7.5
Early endosome	6.0–6.5
Late endosome	5.0–6.0
Lysosome	4.5–5.0
Golgi	6.4
Tumour, extracellular	7.2–6.5
Stomach	1.0–3.5
Duodenum	4.8–8.2
Liver	7.4

**Table 3:** Examples of thermo-responsive polymers.

Polymer	Type	CST, °C	Reference
Poly(*N*-n-propylacrylamide) (PNNPAM)	LCST	10	[73]
Poly(ethylene oxide) (PEO)	UCST	230	[74]
Poly(*N*-isopropylacrylamide) (PNIPAM)	LCST	32	[75]
Poly(vinyl methyl ether) (PVME)	LCST	−15, −25	[76,77]
Poly(2-isopropyl-2-oxazoline) (PiPOx)	LCST	36	[78]
Poly(methyl methacrylate) (PMMA)	UCST	87 or above	[79]
Poly(2-hydroxypropylacrylate) (PHPA)	LCST	30–60	[80]

## Abbreviations

Camptothecin	CPT
Cancer stem cells	CSCs
Chitosan	CS
Doxorubicin	DOX
FTS-hydrazide	FTS-H
Graphene oxide	GO
Lower critical solution temperature	LCST
Magnetic nanoparticles	MNPs
Methoxy(polyethylene glycol)	mPEG
*N*-(2-hydroxypropyl)methacrylamide)	HPMA
*N*-(3-aminopropyl) methacrylamide hydrochloride	APMA
Paclitaxel	PTX
Photodynamic therapy	PDT
Pirarubicin	PIR
Polyacrylic acid	PAA
Poly (d,l-lactide-co-glycolide)	PLGA
Poly(ethylene oxide)	PEO
Poly (methacrylic acid)	PMAA
Poly(methyl methacrylate)	PMMA
Poly(phenylene vinylene)s	PPVs
Poly(*N*,*N*-dimethyl aminoethyl methacrylamide)	PDEAEMA
Poly(*N*-isopropylacrylamide)	PNIPAM
Poly(*N*-isopropylacrylamide-acrylamide-allylamine)	PMNPs
Poly(*N*-n-propylacrylamide)	PNNPAM
Poly(propylene)	PP
Poly(vinyl methyl ether)	PVME
Poly(2-hydroxypropylacrylate)	PHPA
Poly(2-isopropyl-2-oxazoline)	PiPOx
Single photon emission computerized tomography	SPECT
Ultraviolet	UV
Upper critical solution temperature	UCST
